# Pathogenic and therapeutic roles of extracellular vesicles in sepsis

**DOI:** 10.3389/fimmu.2025.1535427

**Published:** 2025-02-04

**Authors:** Benshuai You, Yang Yang, Jing Wei, Chenglin Zhou, Surong Dong

**Affiliations:** ^1^ Clinical Laboratory Center, The Affiliated Taizhou People’s Hospital of Nanjing Medical University, Taizhou, Jiangsu, China; ^2^ Department of Obstetrics and Gynecology, The Affiliated Taizhou People’s Hospital of Nanjing Medical University, Taizhou, Jiangsu, China

**Keywords:** sepsis, extracellular vesicles, intercellular communication, treatment, engineering strategies

## Abstract

Sepsis is a systemic injury resulting in vascular dysfunction, which can lead to multiple organ dysfunction, even shock and death. Extracellular vesicles (EVs) released by mammalian cells and bacteria have been shown to play important roles in intercellular communication and progression of various diseases. In past decades, the functional role of EVs in sepsis and its complications has been well explored. EVs are one of the paracrine components of cells. By delivering bioactive materials, EVs can promote immune responses, particularly the development of inflammation. In addition, EVs can serve as beneficial tools for delivering therapeutic cargos. In this review, we discuss the dual role of EVs in the progression and treatment of sepsis, exploring their intricate involvement in both inflammation and tissue repair processes. Specifically, the remarkable role of engineered strategies based on EVs in the treatment of sepsis is highlighted. The engineering EVs-mediated drug delivery and release strategies offer broad prospects for the effective treatment of sepsis. EVs-based approaches provide a novel avenue for diagnosing sepsis and offer opportunities for more precise intervention.

## 1 Introduction

Sepsis is a medical emergency without a clearly defined treatment method. It is one of the most common causes of death, closely associated with uncontrolled systemic inflammation (1, 2). The first modern definition of sepsis, proposed in 1992, described it as an excessive inflammatory response to infection, identified by the presence of the systemic inflammatory response syndrome (SIRS) (3). SIRS is characterized by two or more abnormalities in temperature, heart rate, respiratory rate, or white blood cell count. The pathobiology of sepsis is characterized by simultaneous inflammation and impaired immune function, along with significant microvascular damage (4). A pathogen is identified in about 60 to 70% of cases (5). The most frequent cause is bacterial infection, either gram-positive or gram-negative, followed by fungal or viral infections. In 2017, there were reported 11 million sepsis-related deaths globally, accounting for 19.7% of all global deaths (6). Over the years, sepsis has remained a highly fatal condition with suboptimal diagnostic accuracy and limited therapeutic decision-making capabilities. The outcome of the pathobiological process of sepsis is organ damage, frequently leading to multiorgan failure. Sepsis is often accompanied by widespread acute lung injury (ALI), acute kidney injury (AKI), liver injury and myocardial injury, among other life-threatening complications, for which timely and effective pharmacological interventions are still lacking (7–9). Therefore, studying the pathogenesis and intervention methods of sepsis is of great significance for improving the prognosis of sepsis.

**Figure 1:**
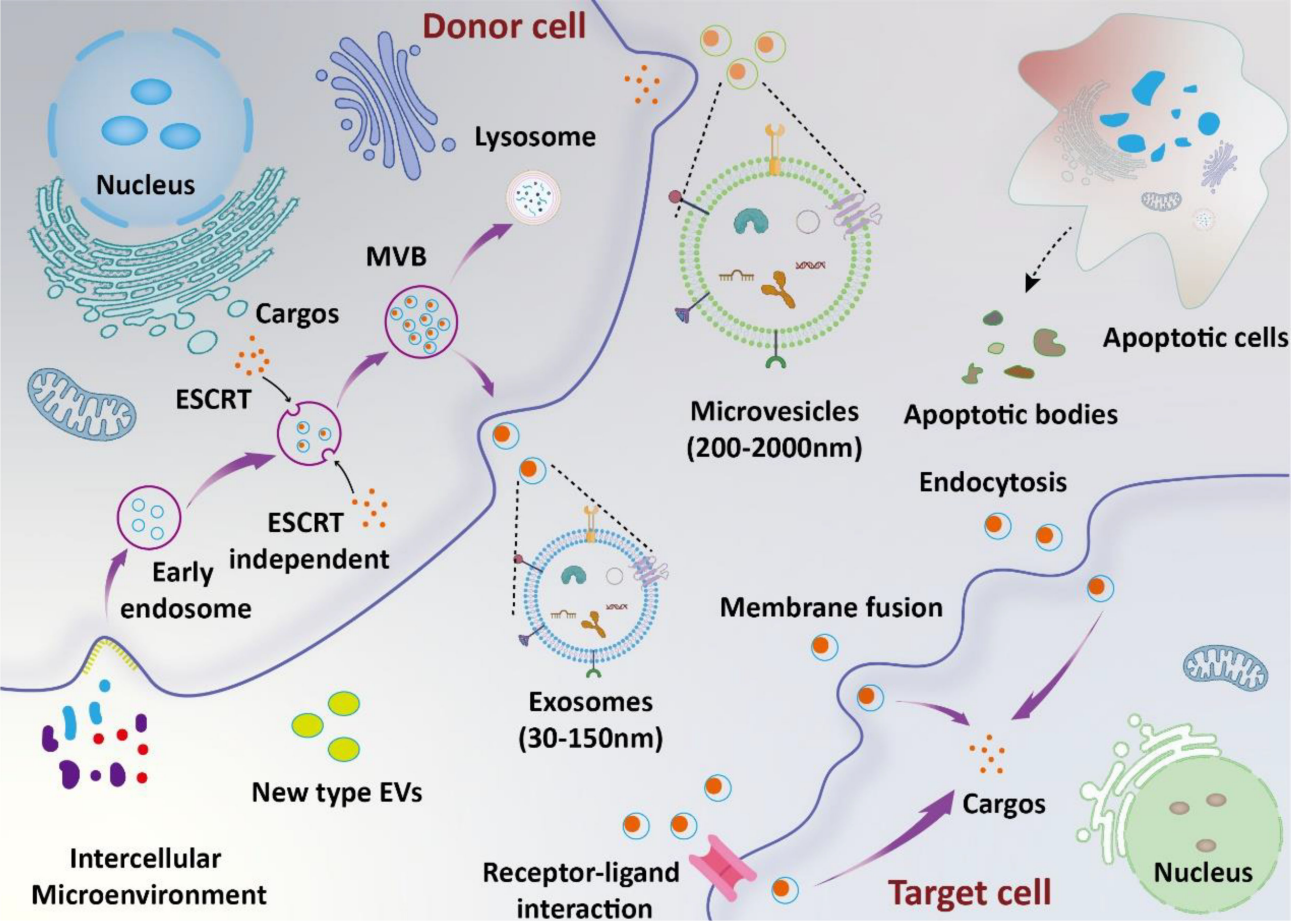
Biosynthesis and function of EVs. The biosynthesis of exosomes involves several stages, including lipid raft-mediated endocytosis, the formation of multivesicular bodies (MVBs), and exosome release. Microvesicles are formed through the direct budding of the cell membrane. Apoptotic bodies are produced by apoptotic cells. Extracellular vesicles (EVs) deliver their cargos into target cells through mechanisms such as ligand-receptor interaction, endocytosis, and direct fusion with the plasma membrane.

Extracellular vesicles (EVs) are small vesicles secreted by various cells, consisting of a double-layered membrane structure (10). According to the recommendation of the International Society for Extracellular Vesicles (ISEV), EVs could be defined into subtypes based on physical characteristics (e.g., size and density), biochemical composition, descriptions of conditions, or cell of origin (11). In previous studies, EVs have been commonly referred to as exosomes, microvesicles, and apoptotic bodies (12). In recent years, new types of EVs, such as retractosomes and migrasomes, have been identified (13, 14). However, their biogenesis and physiological functions remain largely unknown. In this manuscript, we use the names of EVs as they appear in their original articles. The formation and release of different types of EVs are illustrated in the **Figure 1**. They contain a variety of bioactive components such as proteins, RNA, lipids, etc., and play significant roles in intracellular and intercellular communication (15–17). The research indicates that EVs primarily deliver their cargo to target cells via ligand-receptor interactions, endocytosis, and direct fusion with the plasma membrane (18). Recent studies have shown that EVs are deeply involved in the occurrence and development of sepsis (19). On one hand, EVs secreted between cells under disease conditions promote sepsis-induced inflammatory storms and organ damage by interacting with recipient cells. On the other hand, exogenous EVs can deliver therapeutic molecules to inhibit systemic inflammation and promote tissue function recovery, thus alleviating disease progression.

In this review, we will focus on the role of EVs as messengers and therapeutic tools in the context of sepsis, with particular attention to the advancements in engineering modifications to enhance the efficacy of EVs. A deeper understanding of the potential of EVs in the pathogenesis and therapeutic interventions of sepsis can contribute to the timely resolution of this medical challenge.

## 2 EVs-mediated intercellular communication in sepsis

EVs are rich in various bioactive components and play a significant role in intercellular communication. Current research indicates that EVs participate in the occurrence and progression of sepsis through multiple pathways (20). Increased plasma EVs levels correlate with the severity of organ failure and can serve as predictors of mortality in patients with sepsis (21). EVs could serve as an innovative mode of intercellular communication in the process of sepsis. In this section, we focus on the changes in cargo of EVs in sepsis and how EVs from different sources participate in various pathological processes and organ dysfunction in sepsis.

Circulating EVs in the body, which carry biomarkers and mediators of sepsis, have garnered interest. EVs containing an abundance of cytokines and chemokines play crucial roles in T cell differentiation, proliferation, and chemotaxis throughout the course of sepsis (22). EVs derived from individuals experiencing septic shock carry miRNAs and mRNAs associated with pathogenic pathways, such as inflammatory response, oxidative stress, and cell cycle regulation (23). Circulating EVs notably exacerbate the inflammatory response to sepsis in both serum and lung tissue by enhancing the production of proinflammatory factors such as tumor necrosis factor-α (TNF-α), interleukin-6 (IL-6), and interleukin-1β (IL-1β) (24). Compared with healthy individuals, 354 proteins, 195 mRNAs, 82 lncRNAs, and 55 miRNAs were found to be differentially expressed in serum exosomes from septic patients (25). Furthermore, pretreating septic mice with serum exosomes obtained from mice undergoing cecal ligation and puncture (CLP) notably suppressed the expression of proinflammatory cytokines and mitigated tissue damage. Caspase-1, a member of the cysteine protease family, is essential for regulating inflammatory responses (26). miR-126 could enhance the outcomes of acute lung injury induced by lipopolysaccharide and sepsis induced by cecal ligation and puncture (27). Increased caspase-1 activity and decreased levels of miR-126 in circulating extracellular vesicles are correlated with organ failure and mortality in sepsis (28). The combination of serum exosomal miR-483-3p and let-7d-3p showed diagnostic value for sepsis (29). The multi-omics analyses above indicate that EVs may play a significant role in the progression of sepsis. During sepsis, endothelial-derived microvesicles exacerbate endothelial inflammation by enhancing the adhesion between neutrophils and the endothelium and facilitating the release of neutrophil extracellular traps that contain citrullinated histones and myeloperoxidase (30). Endothelial EVs induce a pro-inflammatory phenotype in monocytes through aberrant expression of miR-99a and miR-99b, thus inhibiting mTOR expression (31). Lipinski et al. demonstrated that inflammasome-activated macrophages-derived EVs were able to transmit a robust IL-1β-dependent inflammatory response in sepsis mice (32). The shuttle of EVs is beneficial for the transmission of inflammatory signals in sepsis, thereby accelerating the progression of sepsis. The development of EVs-based detection technologies may provide valuable diagnostic and therapeutic insights for the ongoing battle against sepsis.

**Figure 2:**
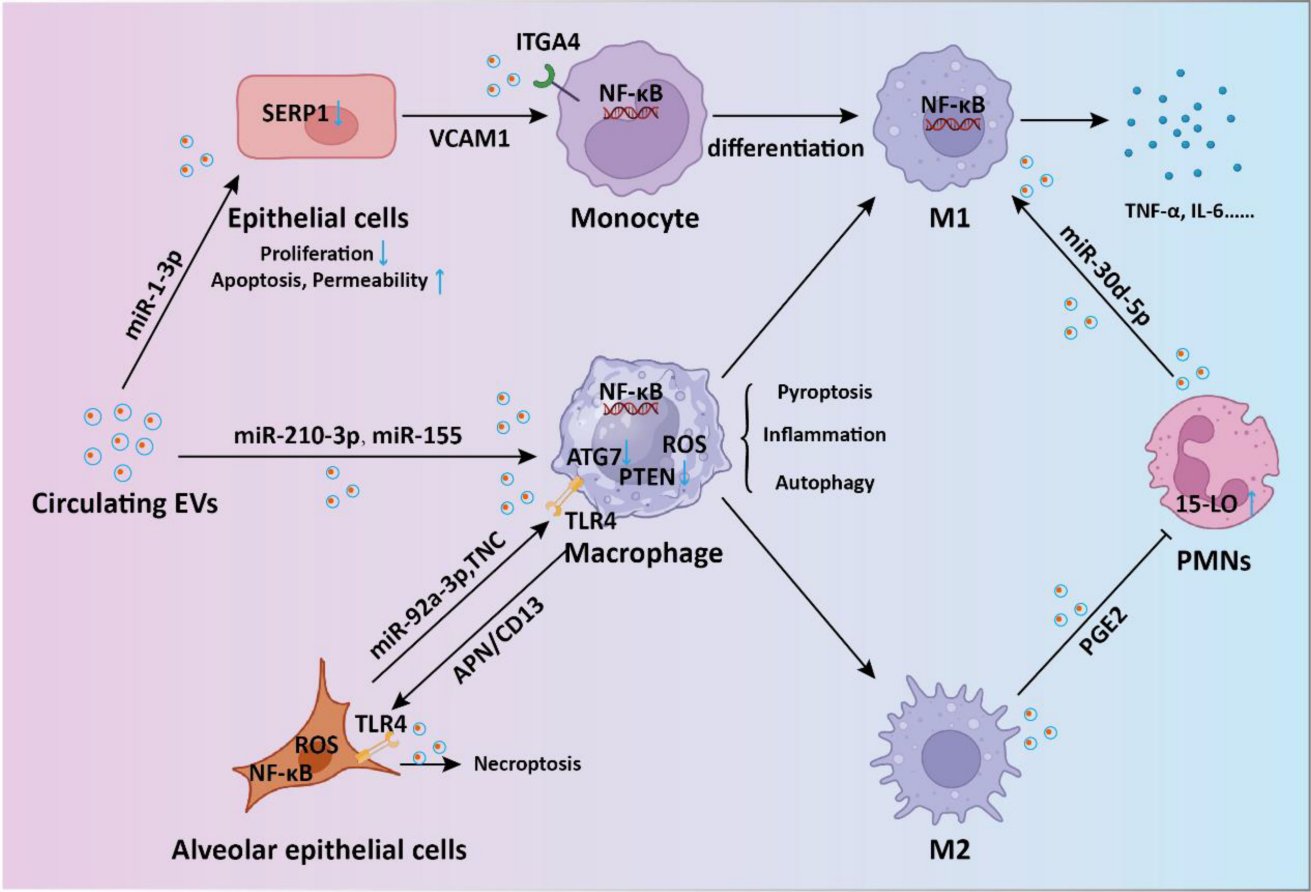
The intercellular communication mediated by EVs in sepsis-induced acute lung injury. During the process of sepsis-induced acute lung injury, EVs-mediated shuttling of miRNA and proteins regulates the signaling between endothelial cells, macrophages, monocytes, and neutrophils, promoting inflammatory responses and thereby accelerating lung injury.

Sepsis-induced ALI stands as a critical complication and the primary contributor to mortality (33). Recent findings suggest that overactivation of macrophages could lead to detrimental lung inflammation implicated in sepsis-induced ALI (34). During sepsis, endothelial cell-derived EVs (EC-EVs) with heightened levels of vascular cell adhesion molecule 1 (VCAM1) stimulate the NF-κB pathway upon binding to integrin subunit alpha 4 (ITGA4) receptors on monocytes (35). This interaction modulates monocyte differentiation, shifting them toward a proinflammatory M1 macrophage phenotype, thereby promoting sepsis-related ALI. EVs originating from CD4^+^ T cells delivered diacylglycerol kinase kappa (DGKK) to induce apoptotic cell death, oxidative damage, and inflammation in alveolar epithelial cells, exerting toxic effects (36). The messenger role of EVs in mediating the crosstalk between alveolar epithelial cells and alveolar macrophages is crucial for ALI progression. Liu et al. showed alveolar epithelial cells-derived exosomes could deliver miR-92a-3p to activate macrophages, resulting in activation of the NF-κB pathway and downregulation of PTEN expression along with pulmonary inflammation (37). Gong et al. demonstrated that alveolar epithelial cells derived exosomes containing tenascin-C (TNC) bind to Toll-like receptor 4 (TLR4) on macrophages, leading to increased production of reactive oxygen species (ROS) and subsequent activation of the NF-κB pathway (38). The processes ultimately result in macrophage pyroptosis, thus exacerbating the release of inflammatory cytokines. On the other hand, exosomal APN/CD13 derived from macrophages regulates necroptosis in lung epithelial cells by interacting with the cell surface receptor TLR4, thereby triggering ROS production, mitochondrial dysfunction, and NF-κB activation (39). Polymorphonuclear neutrophils (PMNs) are pivotal in sepsis-related ALI (40). Jiao et al. revealed that exosomal miR-30d-5p from PMNs contributed to sepsis-related ALI by inducing M1 macrophage polarization and triggering macrophage pyroptosis through the activation of NF-κB signaling (41). In another study, M2 macrophage-derived exosomes(M2-Exos) were able to inhibit PMN migration and neutrophil extracellular trap (NET) formation, thus alleviate lung injury and reduce mortality in a sepsis mouse model (42). Mechanistically, the secreted prostaglandin E2 (PGE2) contained within M2-Exos could bind to the EP4 receptor expressed on PMNs, thereby upregulating the expression of 15-LO in PMNs. In addition to EVs in the pulmonary microenvironment, circulating EVs are also involved in the progression of sepsis-induced ALI. Plasma extracellular vesicles deliver miR-210-3p, targeting ATG7 to enhance sepsis-induced ALI by modulating autophagy and triggering inflammation in macrophages (43). In addition, serum exosomes could transfer miR-155 to macrophages, triggering the activation of nuclear factor κB (NF-κB) and prompting the secretion of TNF-α and IL-6 in ALI (44). Sepsis plasma-derived exosomal miR-1-3p was able to induce endothelial cell dysfunction by targeting SERP1 (45). This inhibition results in decreased cell proliferation, increased apoptosis, cytoskeleton contraction, heightened monolayer endothelial cell permeability, and membrane injury. Together, these investigations highlight the significance of circulating EVs released into the bloodstream as crucial mediators of septic lung injury through the transport of cargo via EVs (**Figure 2**).

Sepsis-induced AKI and cardiomyopathy are common complications of sepsis. Sepsis-associated AKI is a critical complication with high morbidity and mortality rates among critically ill patients (46). Sepsis-induced cardiomyopathy is prevalent among septic patients, and is distinguished by a decreased ejection fraction (47). Studies have shown that EVs are involved in kidney and myocardial damage during the progression of sepsis. Renal tubular epithelial cells (RTECs) are the predominant cell type in the kidney and have a vital role in pathological renal injuries (48). Plasma EVs in circulation primarily derive from platelets and can contribute to organ dysfunction during sepsis. EVs loaded with ARF6 activate ERK/Smad3/p53 signaling in RTECs, exacerbating sepsis-induced AKI (49). M1 and M2 macrophages exosomal miR-93-5p could regulate the TXNIP expression directly to influence the pyroptosis in RTECs (50). During sepsis-induced cardiomyopathy, endothelial HSPA12B participates in regulating macrophage pro-inflammatory responses via EVs (51). miRNAs originating from extracellular vesicles derived from neutrophils play a significant role in the progression of septic disease severity towards cardiomyopathy. Ye et al. found that 38 miRNAs showed differentially expression between the septic cardiomyopathy and without cardiomyopathy patients, especially the independent predictor potential of miR-150-5p (52). In septic myocardial depression, circulating EVs promoted the pyroptosis of cardiomyocyte through miR-885-5p via HMBOX1 (53). Septic exosomes were found to be highly enriched with ROS, which can be transferred to endothelial cells. This transfer leads to the formation of podosome clusters in target endothelial cells, resulting in the relocation of ZO-1, vascular leakage, and cardiac dysfunction (54). Overall, these studies emphasize the crucial roles of EVs in septic development towards cardiomyopathy.

In addition to the aforementioned complications, liver injury is also a common organ dysfunction in sepsis, manifesting in around 50% of septic patients (55). EVs have been shown to be involved in sepsis induced liver injury. High mobility group box protein 1 (HMGB1) serves as a prototypical damage-associated molecular pattern (DAMP) molecule, exerting cytotoxic effects that result in cell death and tissue injury (56). Utilizing transferrin-mediated endocytosis, macrophage-derived EVs transport HMGB1 to target cells, inducing hepatocyte pyroptosis through the activation of NLRP3 inflammasomes (57). Platelet-derived exosomes deliver HMGB1 and/or miR-15b-5p and miR-378a-3p to induce NET formation and subsequent organ injury by the activation of Akt/mTOR autophagy pathway (58). Yang and colleagues discovered that macrophages possess the capability to absorb extracellular lactate using monocarboxylate transporters (MCTs), thus facilitating HMGB1 lactylation through a p300/CBP-dependent pathway (59). The lactylated/acetylated HMGB1 is subsequently discharged from macrophages through exosome secretion, consequently enhancing endothelium permeability.

## 3 The beneficial effects of EVs in sepsis treatment

Patients with severe sepsis often exhibit a heightened inflammatory response, which is a key factor leading to multiple organ dysfunction (60). Therefore, anti-inflammatory intervention has been considered an effective approach in the treatment of sepsis. Mesenchymal stem cells (MSC) are the most common source of EVs used in the treatment of sepsis. MSC-derived exosomes could mitigate the destructive effects of sepsis-induced inflammation by reducing inflammatory factors and tissue damage, leading to decreased levels of cytokines such as IL-6, IL-1β, and TNF-α (61). miRNAs packaged within EVs have been implicated in the pathophysiology of sepsis. Sun et al. revealed that MSC-derived exosomes containing miR-27b suppressed sepsis progression by reducing the production of pro-inflammatory cytokines (62). This effect was achieved through the downregulation of JMJD3 and inhibition of the NF-κB signaling pathway in bone marrow-derived macrophages. Similarly, adipose MSC-derived EVs (ADSC-EVs) were able to attenuate sepsis-induced inflammation by modulating the Notch/miR-148a-3p signaling pathway, which also decreased macrophage polarization to M1 (63). In addition to inflammation response, oxidative stress also deeply participates in the progression of sepsis. ADSC exosomes exerted protective effect in sepsis by relieving inflammatory cytokines storm and oxidative stress (64). Mechanistically, ADSC exosomes could regulate Nrf2/HO-1 axis in macrophages, thus promoting the polarization of macrophages from the M1 to M2 phenotype. Bone marrow MSC-derived EVs (BMSC-EVs) therapy enhanced survival, decreased sepsis-induced inflammation, reduced pulmonary capillary permeability, and improved liver and kidney function via delivery of miR-21a-5p (65). Therefore, the intervention effect of EVs on inflammatory response is a key factor in the treatment of sepsis. MSC-derived apoptotic vesicles (apoVs) could improve survival and alleviate multiple organ dysfunction in septic mice (66). Mechanistically, it is discovered that apoVs infused through the tail vein predominantly gather in the bone marrow of septic mice through electrostatic interactions with positively charged NET. Additionally, apoVs induce a shift in neutrophils from NETosis to apoptosis via the apoV-Fas ligand (FasL)-activated Fas pathway. Pyroptosis is an inflammatory programmed cell death process (67). EVs derived from pyroptotic MSC show promising benefits in the treatment of sepsis. Huang et al. showed that pyroptotic EVs specifically express pyroptotic maker ASC and bind to B cells to repress cell death by repressing Toll-like receptor 4 (68). The pyroptotic EVs were found to alleviate inflammatory responses and improve the survival rate of mice with sepsis.

**Figure 3:**
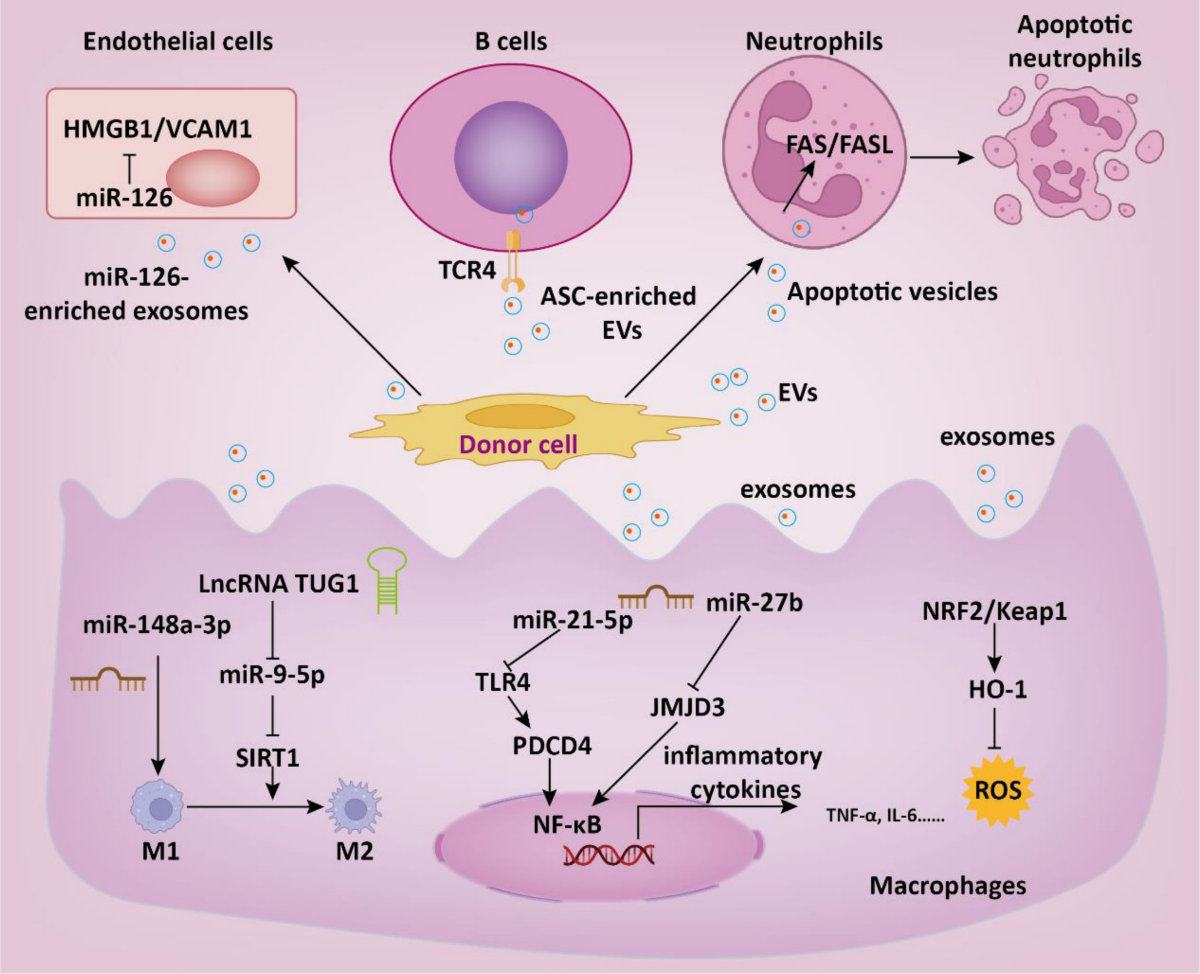
The role of EVs in the therapeutic intervention of sepsis. EVs regulate multiple intracellular signaling pathways in target cells by delivering functional cargoes such as miRNA, lncRNA, proteins, etc., thereby suppressing inflammatory responses, reducing thrombosis formation, and consequently slowing down the progression of sepsis.

In addition to MSC, EVs derived from other cell sources have also been shown to have anti-sepsis effects. Zhou and colleagues demonstrated that endothelial progenitor cells derived exosomes may prevent microvascular dysfunction and improve sepsis outcomes potentially through the delivery of miR-126 (69). Mechanistically, miR-126-5p and 3p inhibited the levels of lipopolysaccharide (LPS)-induced HMGB1 and VCAM1, respectively, in human microvascular endothelial cells (HMVECs). Endothelial progenitor cell-derived EVs transferred lncRNA TUG1, facilitating M2 macrophage polarization by disrupting miR-9-5p-mediated inhibition of SIRT1 (70). Septic shock is marked by profound systemic inflammation, heightened coagulation activation, and impaired fibrinolysis, culminating in disseminated intravascular coagulation (DIC) (4). Cointe et al. demonstrated that elevated levels of platelet granule content (PGC) in granulocyte-derived microvesicles (Gran-MVs) reduce thrombus formation and enhance survival, indicating a protective function of Gran-MVs in sepsis (71). DIC is a common severe complication of sepsis, currently lacking effective treatment methods. In another study, Bao and colleagues demonstrated that neutrophils mitigate sepsis-associated coagulopathy via EVs containing superoxide dismutase 2 (72). Mechanistic investigations revealed that superoxide dismutase 2 is essential for inducing neutrophil anti-thrombotic function by preventing endothelial reactive oxygen species accumulation and relieving endothelial dysfunction (**Figure 3**).

EVs derived from multiple sources have been used for the intervention of sepsis-induced ALI. A study compared the protective effects of exosomes derived from adipose tissue, bone marrow, and umbilical cord on sepsis-induced ALI (73). The results showed that all three types of exosomes effectively downregulated sepsis-induced macrophage glycolysis and inflammation, improved lung pathological damage, and enhanced the survival rate of septic mice. Research has found that ADSC-derived exosomes deliver miR-125b-5p to inhibit Keap1, thereby upregulating Nrf2/GPX4, reducing ferroptosis in microvascular endothelial cells, ultimately alleviating lung tissue damage and lowering mortality rates (74). Additionally, ADSC-derived exosomes could suppress macrophage aggregation and IL-27 secretion in lung tissues, leading to reduced pulmonary edema and pulmonary vascular leakage (75). The excessive expression of SAA1 in BMSC-derived exosomes suppressed lung injury by reducing the levels of endotoxin, TNF-α, and IL-6 induced by CLP or LPS (76). Exosomes from endothelial progenitor cells were able to enhance the prognosis of lipopolysaccharide-induced ALI by delivering miR-126 to the injured alveoli (77). Endoplasmic reticulum (ER) stress exacerbates sepsis-induced ALI. Chiang et al. showed that human placental MSC-derived exosomes could alleviate lipopolysaccharide-induced ER stress, inflammation, and lung injury in mice (78).

Some studies have explored the beneficial effects of EVs in sepsis-related AKI. The most common contributing factor for AKI among ICU patients is septic shock, accounting for approximately 47.5%, and is associated with a high in-hospital mortality rate (79). Traditional methods to address septic shock, such as fluid administration, antibiotic use, and vasopressors, have not led to improved outcomes in cases of sepsis-related AKI (80). Therefore, EVs-based biologic therapy has significant clinical value in improving sepsis-associated AKI. BMSC-derived exosomes could alleviate inflammation and apoptosis in sepsis-associated AKI through activating autophagy (81). ADSC-derived exosomes were able to protect against sepsis induced inflammation and apoptosis in AKI via activation of SIRT1 signaling pathway (82). Zhang et al. found that endothelial progenitor cells-derived exosomes could release miR-21-5p and miR-93-5p to mitigate sepsis-induced AKI by suppressing RUNX1 expression and KDM6B/H3K27me3/TNF-α axis, thereby enhancing renal function and ameliorating renal tissue pathology (83, 84). This intervention attenuated serum inflammatory response and diminishes apoptosis and oxidative stress in renal tissues.

**Figure 4:**
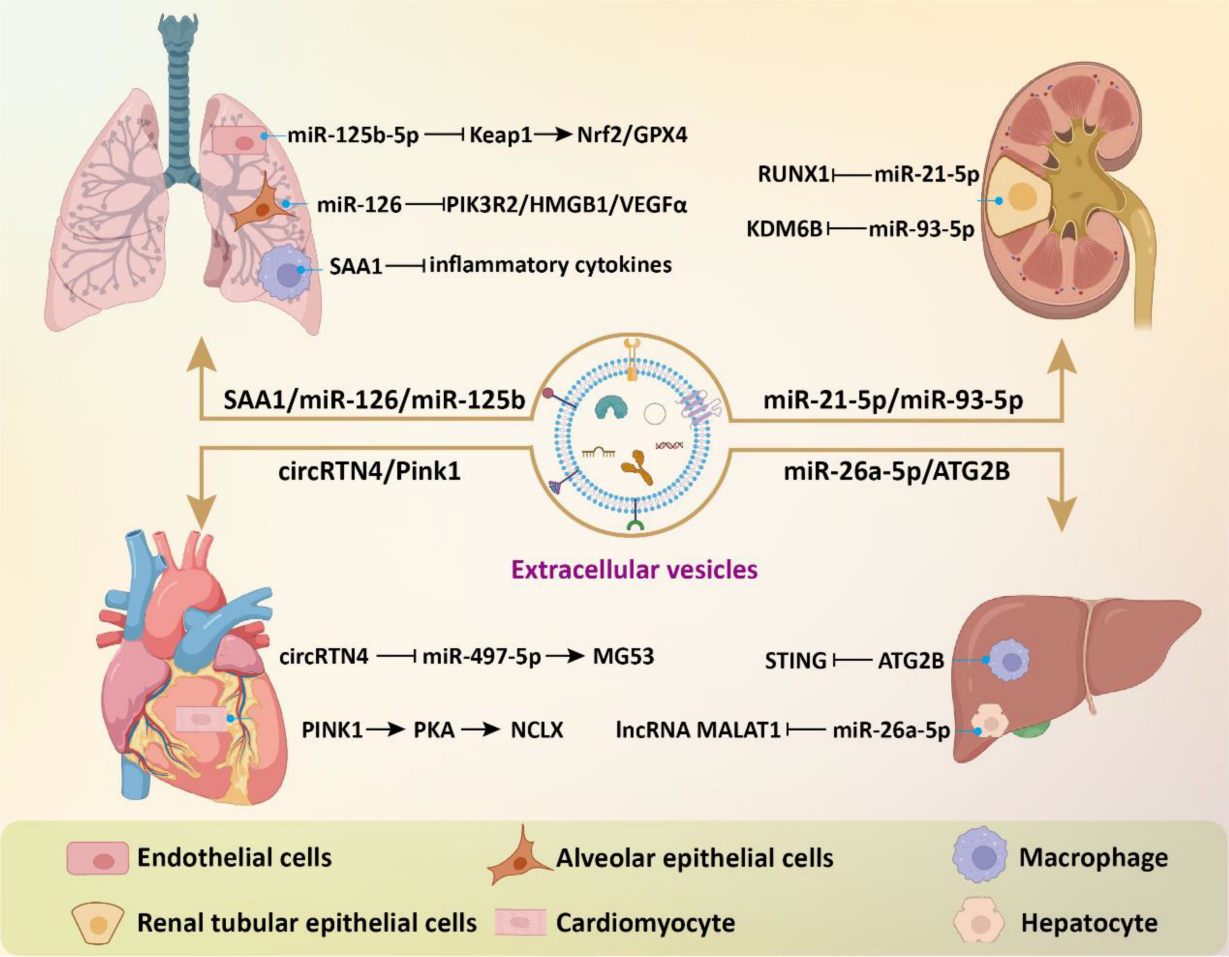
The beneficial effects of EVs in sepsis-related complications. EVs modulate intracellular signaling in effector cells of target organs by delivering bioactive components, thereby inhibiting inflammatory responses, reducing cell death and oxidative stress, and consequently alleviating tissue damage.

EVs have also shown great potential for application in sepsis-induced myocardial and liver injuries. In a study, Li et al. found that MSC-derived exosomes prevented sepsis-induced myocardial injury by delivering circRTN4 to inhibit miR-497-5p, thereby upregulating MG53 in cardiomyocytes (85). In another research, Zhou and colleagues demonstrated that umbilical cord MSC-derived exosomes transported Pink1 mRNA to recipient cardiomyocytes, enhancing PINK1 expression (86). This process helped mitigate mitochondrial calcium overload in sepsis by restoring mitochondrial calcium efflux through the PINK1-PKA-NCLX axis. For acute liver injury, treatment with MSC-EVs led to a notable decrease in the expression of multiple glycolysis-related enzymes and suppressed glycolytic flux by inhibiting the expression of hypoxia-inducible factor 1α (HIF-1α), thereby effectively dampening macrophage inflammatory responses (87). MSC-derived exosomal miR-26a-5p could significantly protect against hepatocyte death and liver injury caused by sepsis through targeting lncRNA MALAT1 (88). LPS-pretreated BMSC-derived exosomes (L-Exo) notably alleviated septic liver injury induced by cecal ligation and puncture and suppressed macrophage STING signaling (89). Mechanistically, L-Exo inhibited macrophage STING signaling by delivering ATG2B to promote mitophagy and prevent the release of mtDNA into the cytosol. In addition, the combination of MSC and hepatocyte-derived exosomes with imipenem was able to improve the inflammatory response and liver damage in sepsis, thus increasing survival rates (90). Compared to treating with a single drug, combination therapy offers an alternative strategy (**Figure 4**).

## 4 Engineering methods for improving treatment effect of EVs in sepsis

EVs hold promise in the treatment of sepsis, yet they suffer from limitations such as low therapeutic cargos and weak targeting. To overcome these shortcomings, various engineering strategies for modifying EVs have been developed. In this section, we introduce genetic and non-genetic methods for remodeling EVs and focus on the enhanced role of engineered EVs in sepsis therapy.

To increase the functional cargos within EVs, exogenous manipulation of donor cells or isolated EVs is employed. Li et al. discovered that tumor cell-secreted exosomes produced after LPS treatment are more effective in improving sepsis compared to normal secretory exosomes due to the presence of protective miRNAs (91). To mimic these exosomes, they developed exosome mimics by incorporating the mentioned microRNAs into hyaluronic acid-polyethylenimine nanoparticles. These exosome mimics, with specific miRNA compositions, mitigate sepsis in mice and cynomolgus monkeys, suggesting that biomimetic simulation of tumor-suppressive exosomes may offer a promising therapeutic approach for treating sepsis and cytokine-storm-related conditions. MicroRNA-146a is a widely reported negative immunoregulatory small noncoding RNA (92). In another study, researchers developed an engineered macrophages-derived apoEVs based multifunctional agents for sepsis treatment (93). ApoEVs, engineered with mesoporous silica nanoparticles carrying miR-146a, captured iron and neutralized α-toxin using their natural membrane, and modulated inflammation by releasing miR-146a in phagocytes. These engineered apoEVs exhibited a high capacity for capturing toxins and iron, ultimately providing protection against sepsis associated with high iron loads. Dysregulation of M1 macrophage polarization in sepsis leads to serious inflammation. In a study, exosomes were modified through introduction of super-repressor IκB (srIκB) by an optogenetically engineered exosome system (94). In septic mouse models, intraperitoneal administration of purified srIκB-loaded exosomes alleviates mortality and systemic inflammation, potentially by blocking the translocation of NF-κB into the nucleus in neutrophils and monocytes. In another study, EVs derived from immortalized bone marrow-derived macrophages are loaded with siRNA targeting the chemokine receptor CCR2 for targeted drug delivery (95). These EVs containing siCCR2 not only inhibited the chemotaxis of inflammatory monocytes/macrophages but also alleviated septic symptoms in mice by reducing the mobilization of splenic inflammatory monocytes and attenuating the subsequent serum cytokine storm.

In addition to direct manipulation of isolated EVs, modifying the cell culture environment or implementing exogenous pretreatment is an effective strategy to enhance the therapeutic efficacy of EVs. EVs derived from pretreated MSC have demonstrated significant promise in addressing diverse inflammatory conditions. Pre-treated with LPS, BMSC-EVs significantly alleviated septic liver injury induced by cecal ligation and puncture through the inhibition of macrophage STING signaling (89). These BMSC-EVs delivered autophagy-related protein 2 homolog B (ATG2B) for promoting mitophagy and suppressing the release of mtDNA into the cytosol. Additionally, IL-1β-primed MSC-derived exosomes exhibited beneficial effects on macrophage polarization and sepsis (96). These exosomes delivered miR-21 to macrophages, targeting PDCD4 and inducing M2 polarization. These studies underscore a novel basis for inducing immunomodulation for the therapeutic application of MSC-EVs in sepsis. Hypoxia triggers the upregulation of genes associated with proliferation and angiogenesis in MSC, enhancing their differentiation capacity and altering the content of EVs (97–99). Cao et al. showed that hypoxia-preconditioned exosomes derived from ADSC exhibited greater suppression of renal vascular leakage and reduced renal dysfunction compared to control exosomes, thereby improving the survival rate of septic mice (100). These protective effects could be partially attributed to the renal microvascular protective role of exosome-derived circ_0001295.

Aside from the direct administration of exogenous EVs, increased serum EVs induced by remote ischemic preconditioning (RIPC) have been shown to be beneficial in treating sepsis. Pan et al. demonstrated that RIPC, induced by short periods of ischemia and reperfusion in femoral arteries, confers protective benefits against sepsis-induced AKI through the transportation of miR-21 via serum exosomes (101). Mechanistically, exosomal miR-21 was assimilated into renal tubular epithelial cells and subsequently aimed at the downstream PDCD4/NF-κB and PTEN/AKT pathways, subsequently exerting anti-inflammatory and anti-apoptotic effects. For sepsis induced ALI, RIPC could attenuate pulmonary edema, and inflammatory cell infiltration into lung tissues (102). The elevated expression miR-142-5p in serum EVs could target PTEN to activate the PI3K/Akt signaling pathway, thus alleviating ALI. These studies propose novel approaches for intervening in sepsis and highlight the crucial role of EVs in mediating therapeutic benefits.

The weak targeting ability of EVs is one of the main obstacles hindering their application *in vivo*. Li and colleagues demonstrated that exosomes derived from CD5L lentivirus infection and kidney tubular cell targeting peptide LTH modified fibroblastic reticular cells showed enhanced binding specificity to kidney tubular cell (103). The CD5L-enriched exosomes selectively adhered to primary kidney tubular cells, fostering kinase PINK-ubiquitin ligase Parkin-mediated mitophagy. This process suppressed pyroptosis and enhanced kidney function by impeding NLRP3 inflammasome activation, ultimately ameliorating the sepsis survival rate. Membrane fusion technology is also widely applied to improve the therapeutic targeting of EVs. Neutrophils are the initial cells recruited to the site of injury during an inflammatory response. Neutrophil membrane was performed on panax ginseng root-derived exosomes following by miR-182-5p electroporation to obtain engineered exosomes (104). Compared with control exosomes, engineered exosomes significantly improved sepsis-induced ALI via target regulation of NOX4/Drp-1/NLRP3 signal pathway.

**Figure 5:**
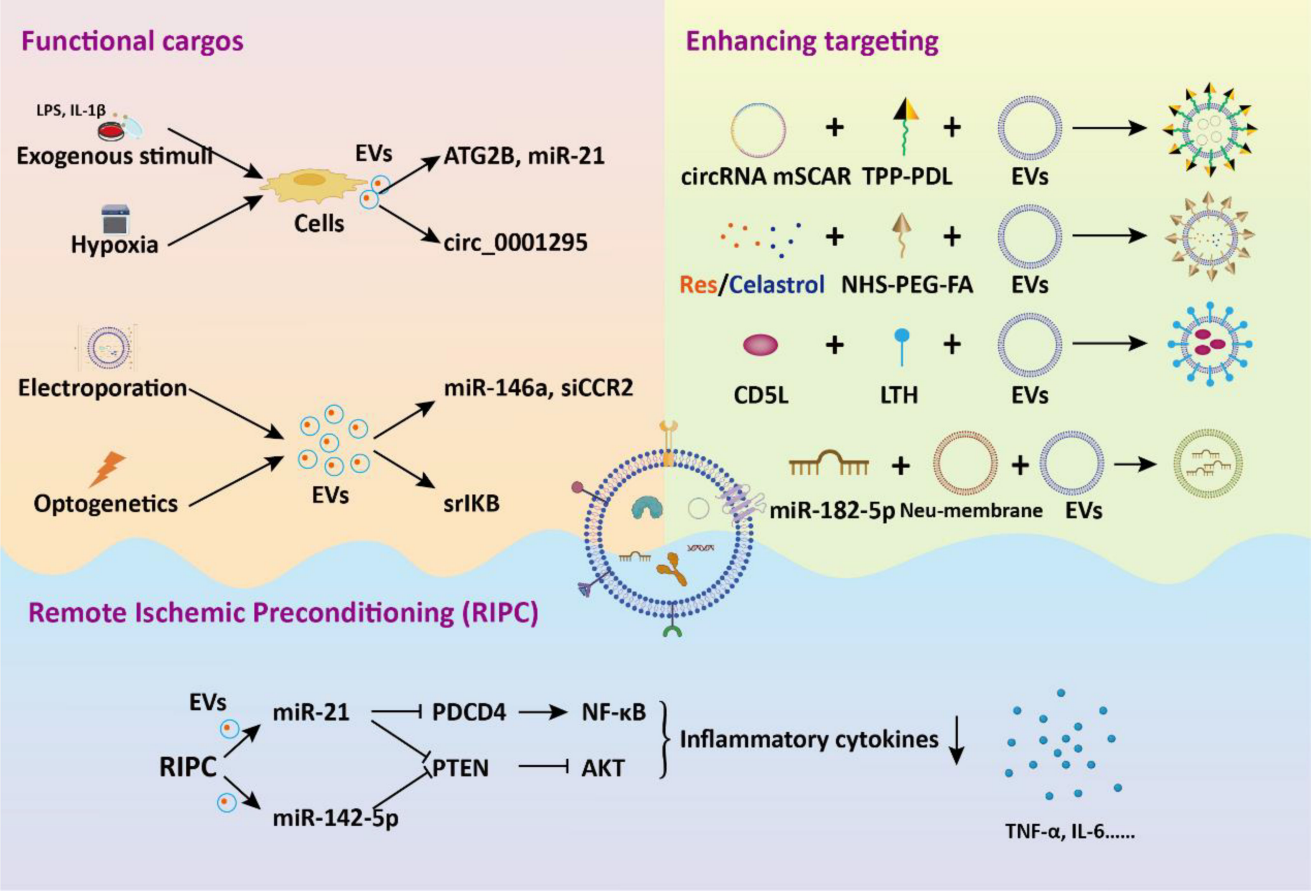
The application of engineered EVs in the treatment of sepsis. Genetic or non-genetic modification strategies enhance the bioactivity or targeting of EVs. These engineered EVs deliver functional cargos to target cells, eliciting biological effects, thereby alleviating sepsis and associated tissue dysfunction.

To target the activated macrophages in sepsis, Fan et al. developed a method involving exosomes encapsulation with circRNA mSCAR and electroporation with TPP-PDL, promoting targeted delivery into mitochondria for macrophage polarization towards the M2 subtype (105). This targeted delivery system attenuates systemic inflammation and reduces mortality in septic mouse and cell models. In order to inhibit overactivated macrophages, Zheng et al. developed folate-functionalized exosomes to target inflammatory macrophages during sepsis in the lungs (106). They found that folate-functionalized exosomes co-loaded with resveratrol and celastrol demonstrated potent anti-inflammatory and immunosuppressive effects against sepsis-stimulated M1 macrophages, thus reducing acute lung injury. These effects were mediated through the regulation of NF-κB and ERK1/2 signaling pathways. Collectively, the strategy of engineered EVs for targeting inflammatory macrophages during sepsis development offers a new avenue for sepsis treatment (**Figure 5**).

## 5 Discussion

Sepsis results from a dysregulated host response to infection, leading to life-threatening organ dysfunction. Despite progress in treatment, the high mortality rate persists due to rising antibiotic resistance and an aging population. Sepsis patients and normal controls exhibit significant differential expression of molecules in serum EVs, including proteins, mRNA, miRNAs, and lncRNAs. An integrative multiomics analysis revealed that components of EVs were linked to cytokine storm, complement and coagulation cascades and endothelial barrier function (25). These studies suggest that EVs could potentially serve as new markers for the diagnosis and monitoring of sepsis. EVs, particularly those derived from MSC, have been shown to promote the restoration of homeostasis in sepsis and alleviate organ damage, facilitating functional recovery. However, the mechanisms by which EVs promote tissue recovery during sepsis have not been fully elucidated. Numerous studies have demonstrated the effectiveness of administration of EVs *in vivo*, there is a lack of detailed elucidation of specific signaling pathways, which introduces many unknown and ambiguous areas for potential clinical translation research.

To further enhance the therapeutic efficacy of EVs and overcome the limitations of natural EVs, various engineering modifications are employed to boost the therapeutic molecular activity, targeting specificity, and biocompatibility of EVs. Collectively, strategies to modify EVs could be a new avenue in developing therapeutics against sepsis and its complications. Currently, the main methods employed involve genetic or non-genetic modifications of parent cells or isolated EVs. Manipulating donor cells to obtain engineered EVs can preserve most of the biophysical characteristics of EVs. However, modification of parent cells may have unforeseen consequences on the biology of EVs, ultimately interfering with EV biogenesis and altering other biological properties. Therefore, an increasing number of studies are focusing on the direct manipulation of EVs. Direct modification of isolated EVs does not alter the biological origin of EVs and allows for the direct impartation of desired biological properties using various methods as needed.

Multiple studies have reported the beneficial effects of EVs, especially those derived from mesenchymal stem cells, in the treatment of sepsis. However, the therapeutic effects of EVs have not yet been widely recognized. A meta-analysis suggests that the treatment of MSC-EVs may be associated with lower mortality rates in septic animal models (107). Nevertheless, there is a lack of standardized data on the dosage, source, and timing of EVs administration for comparison. The therapeutic effects of EVs derived from MSC may vary depending on their source, thus more research is needed to further investigate the complex cargo functionality within EVs (108). Due to the heterogeneous nature of EVs, a more precise definition and classification of EVs subtypes may help enhance the feasibility and therapeutic advantages of EVs in clinical applications. Additionally, there is controversy surrounding the outcomes of interrupting the cascade of sepsis-induced inflammatory responses. More studies are needed to understand the origin and characteristics of EVs, laying the groundwork for comprehending their diversity and complexity. Therefore, it is essential to comprehend the mechanisms of inflammation and develop new treatment approaches to improve the prognosis of sepsis.

In addition to EVs released by mammalian cells during sepsis, numerous studies have highlighted the multifaceted roles of bacterial EVs in this condition. As key mediators of intercellular communication, bacterial EVs impact bacterial pathogenicity, disease mechanisms, and the modulation of the host immune response (109). During sepsis progression, bacterial EVs contribute to pro-inflammatory responses by activating pattern recognition receptors and regulating cytokine release pathways (110). Furthermore, EVs derived from probiotic or commensal bacteria may promote anti-inflammatory responses by modulating anti-inflammatory TLR pathways and cytokine production. For instance, EVs released from probiotic bacteria can enhance macrophage phagocytosis in polymicrobial sepsis through activation of the FPR1/2 pathway (111). Additionally, outer membrane vesicles derived from Escherichia coli Nissle 1917 have been shown to improve immunomodulation and antimicrobial activity in RAW264.7 macrophages (112). These findings underscore the potential of bacterial EVs in modulating host immune cell functions, thus enriching our understanding of EVs as a critical mechanism in both the development and therapeutic management of sepsis.

Overall, EVs-based therapies hold great promise as a potential treatment strategy for sepsis and related complications. EVs also have tremendous potential for early diagnosis and dynamic monitoring of sepsis. However, there is still a long way to go before clinical translation. Extra efforts are needed in standardizing EVs research, understanding their pathogenic mechanisms, and implementing long-term monitoring.
